# Supplementary material for: Regulation of HDL dysfunctionality by phosphatidylethanolamine links poly-unsaturated fatty acids with atherosclerotic cardiovascular diseases
Source: Mol Metab. 2025 Nov 15;103:102281. doi: 10.1016/j.molmet.2025.102281 (PMC12703979; doi:10.1016/j.molmet.2025.102281)
Supplement: Multimedia component 1 [file mmc1.pdf]

SUPPLEMENTAL MATERIALS

REGULATION OF HDL DYSFUNCTIONALITY BY PHOSPHATIDYLETHANOLAMINE LINKS POLY-UNSATURATED FATTY ACIDS WITH ATHEROSCLEROTIC CARDIOVASCULAR DISEASES

Malik Taradeh[1], Lise M. Hardy[1], Veronica Dahik[1], Marie Lhomme[2], Hua Wang[1], Canelle Reydellet[1], Clément Materne[1], Pukar KC[1], Eric Bun[1], Maud Clemessy[1], Jean-Paul Pais-De-Barros[4], Sophie Galier[1], Eric Frisdal[1], Hervé Durand[1], Maharajah Ponnaiah[2], Petra El Khoury[1], Elise F. Villard[1], Philippe Lesnik[1], Antonio Gallo[3], Laurent Kappeler[1], Philippe Giral[3], Eric Bruckert[3], David Masson [5], Maryse Guerin[1], Anatol Kontush[1], Isabelle Guillas[1*] and Wilfried Le Goff[1*]

[1]Sorbonne Université, INSERM, Foundation for Innovation in Cardiometabolism and Nutrition (ICAN), UMR_S1166, F-75013 Paris, France
[2]Foundation for Innovation in Cardiometabolism and Nutrition (IHU ICAN), ICAN I/O data science (MP), ICAN omics (ML), F-75013 Paris, France
[3]Department of Endocrinology and Prevention of Cardiovascular Disease, AP-HP, Hôpital Pitié-Salpêtrière, F-75005 Paris, France
[4]Lipidomic Platform, LNC UMR1231, LabEx LipSTIC, Université de Bourgogne Franche-Comté, Dijon, France
[5]Université Bourgogne Franche-Comté, LNC UMR1231, LabEx LipSTIC, CHU Dijon, Laboratory of Clinical Chemistry, Dijon, France.

[*]IG and WLG contributed equally to this work as co-last authors

Corresponding author
Wilfried Le Goff, *PhD*.
INSERM UMR_S1166
Faculté de médecine Sorbonne Université
91, boulevard de l'Hôpital
75013 Paris
France
Email : wilfried.le_goff@sorbonne-universite.fr

Extended Materials and Methods.

ApoA-I purification.

Total HDL was isolated by sequential ultracentrifugation. The first ultracentrifugation step in Beckman 70 Ti rotor at 45,000 rpm for 24 hours at 15°C at a density of 1.063 g/ml allowed to remove all apo B-containing lipoproteins. Then total HDL was recovered after the second ultracentrifugation step at a density of 1.21 g/ml under the same conditions. Total HDL were extensively dialyzed against ammonium buffer (5 mM, pH 7.4) and lyophilized, resuspended, and delipidated completely with methanol/ether (1:4) at -20°C. Precipitated proteins were dried under nitrogen flux and ApoA-I was purified by ion-exchange chromatography. After protein solubilization in buffer A (20 mM Tris/6 M urea, pH 8.5) at a concentration > 12mg/ml, ApoA-I was loaded on XK26-40 chromatography column and separated at a flow rate of 3 ml/min in Tris-urea buffer by biologic DuoFlow systems (BioRad, France). The purity of individual ApoA-I-containing fractions detected at 280 nm was assessed on 20% denaturing acrylamide gel revealed with Coomassie blue. Pure fractions were pooled and dialyzed against ammonium buffer (20 mM, pH 7.4) and lyophilized[1].

Preparation of rHDL.

rHDL particles were prepared by sodium cholate dialysis as previously described[1]. Briefly, human ApoA-I was mixed with L-α phosphatidylcholine (Soy-PC) without or with either L-α phosphatidylethanolamine (Soy-PE; Avanti Polar Lipids, AL, USA), PE (16:1/20:4), or PE (16:0/20:5; synthesized by ICBMS, Lyon, France), at a molar ratio of 1:90 (ApoA-I: Soy-PC) for Soy-PC rHDL and 1:70:20 (ApoA-I: Soy-PC:PE) for either Soy-PE, PE (16:1/20:4), or PE (16:0/20:5) rHDLs (**Supplemental Table 2**). The required amounts of phospholipids in chloroform were mixed and dried under nitrogen gas. To form micelles, sodium cholate (30 mg/ml) was added to the dried lipids at a molar ratio of 1:1 (sodium cholate: phospholipid) in Tris-buffered saline (TBS, pH 7.4) and vortexed every 15 minutes until the solution became clear. Then ApoA-I was added to the mixture and incubated for 2h at 4C°. Finally, rHDL particles were dialyzed against TBS for 5 days and against PBS for 3 days and stored at -80°C. All the rHDL concentrations were based on their ApoA-I content, which was measured using

Indiko™ Plus clinical chemistry analyzer (Thermo Scientific, US) according to the manufacturer's instructions. Quality control of rHDL was performed using non-denaturing TBE 4–20% gradient polyacrylamide gel electrophoresis and staining with Coomassie Brilliant Blue (**Supplemental Figure 1**)[2].

Lipidomic analysis.

LC/MS-MS was used to characterize the phospholipidome of rHDL particles as previously described[3]. All internal standards were purchased from Avanti Polar Lipids (Alabaster, USA). LC/MS grade or UPLC grade solvents were obtained from Sigma-Aldrich (St Louis, USA) and used without further purification. Plasma lipids were extracted using a modified Folch method[4,5]. Briefly, HDL samples containing 2µg phospholipid supplemented with a mixture of internal standards were mixed with 1600 µL acidified methanol: 0.1N HCl (1:1 v/v) and 800 µL chloroform. The lower organic phase was dried, lipids were reconstituted into 40 µL of LC/MS-compatible solvent and injected into LC/MS-MS system. Lipids species were quantified by LC-ESI/MS-MS using a Prominence UFLC (Shimadzu, Tokyo, Japan) and QTrap 4000 mass spectrometer (AB Sciex, Framingham, USA) equipped with a turbo spray ion source (450°C) combined with an LC20AD HPLC system, a SIL-20AC autosampler (Shimadzu, Kyoto, Japan) and the Analyst 1.5 data acquisition system (AB Sciex, Framingham, MA, USA). Quantification of phospholipids, sphingolipids and neutral lipids was performed in positive ion mode. Sample (4 µl) was injected onto a Kinetex HILIC 2.6 µm (2.1x150 mm) column (Phenomenex, USA). Mobile phases consisted of water and acetonitrile containing ammonium acetate and acetic acid. Lipid species were detected using scheduled multiple reaction monitoring (sMRM). Nitrogen was used as a nebulization and collision gas. Lipids were quantified using 37 calibration curves specific for nine lipid subclasses [phosphatidylcholine (PC), phosphatidylethanolamine (PE), phosphatidylinositol (PI), phosphatidic acid (PA), phosphatidylserine (PS), sphingomyelin (SM), triglycerides (TG), ceramides (Cer) and phosphatidylglycerol (PG)] and up to 12 fatty acid moieties. Highly abundant lipid species which displayed non-linear responses in non-diluted extracts were quantified using a 20-fold diluted sample.

An in-house developed R script was employed to correct for isotopic contribution to MRM signals as adapted from[6].

Atherosclerosis development and injection of reconstituted HDL in mice.

Human transgenic cholesteryl ester transfer protein (CETP) and *Ldlr*$^{-/-}$ (Tg*CETP* x *Ldlr*$^{-/-}$) mice were generated by breeding human CETP transgenic mice [Tg (CETP) 5203Tall/J; Jackson Laboratory] with *Ldlr*$^{-/-}$ (Ldlrtm1Her/J; Jackson Laboratories) mice. Generated Tg*CETP* x *Ldlr*$^{-/-}$ mice were always hemizygous for the human *CETP* transgene. Mice were housed in a conventional animal facility and fed ad libitum a normal chow diet. For the study of atherosclerosis, female Tg*CETP* x *Ldlr*$^{-/-}$ mice who were 9-10 weeks of age (average weight of 22 g) were fed a high-cholesterol diet (HCD) (1.25% cholesterol and 16% cocoa butter, SAFE diet N°CD002510, France) for 8 weeks before the injection of rHDL. Mice under isoflurane anesthesia (2% isoflurane/0.2 L O_2/min) were next retro-orbitally injected with either ARA-PE rHDL (6 mice) or control Soy-PC rHDL (5 mice) at a dose of 15 mg ApoA-I/kg of body weight three times per week for a period of two weeks upon a normal chow diet.

Injection of rHDL was validated through the quantification of plasma levels of human ApoA-I at various time points after a single injection of Soy-PC rHDL (15 mg ApoA-I/kg of body weight) or buffered saline in female Tg*CETP* x *Ldlr*$^{-/-}$mice (**Supplemental Figure 2A**), while the HCD was validated through the quantification of plasma cholesterol levels after 8 weeks of HCD (**Supplemental Figure 2B**). The effectiveness of control Soy-PC rHDL toward atherosclerosis regression was validated in an independent study by injecting female Tg*CETP* x *Ldlr*$^{-/-}$mice with either 15 mg ApoA-I/kg of Soy-PC rHDL or buffered saline three times per week for a period of 4 weeks upon a normal chow diet (**Supplemental Figure 2C and 2D**). Quantification of plasma protein and lipid levels was performed as previously described[4], in blood samples collected in ethylenediaminetetraacetic acid (EDTA)-coated tubes (Microvette, Sarstedt) by retro-orbital bleeding under isoflurane anesthesia (2% isoflurane/0.2 L O_2/min). Plasma samples were stored frozen at -80°C prior use. Mice were sacrificed by cervical dislocation and tissues were collected, snap-frozen and stored at -80°C or fixed in 10% formalin for

further analysis. All procedures were approved and accredited (No. 02458.02) by the French Ministry of Agriculture and were in accordance with the guidelines of the Charles Darwin Ethics Committee on animal experimentation

Antioxidative activity of rHDL.

Antioxidative activity of rHDL (final concentration of 50µg ApoA-I/ml) was evaluated towards reference LDL (final concentration of 0.2 mg cholesterol/ml) isolated from a pool of plasma obtained from healthy subjects by the Etablissement Français du Sang (EFS). LDL was isolated from normolipidemic plasma by isopycnic density gradient ultracentrifugation in a Beckman SW41 Ti rotor at 40,000 rpm for 44 hours at 15°C in optima Beckman XPN-100 ultracentrifuge as described earlier[7]. After ultracentrifugation, the gradient was fractionated in predefined volumes, and five LDL subfractions (LDL1-LDL5) were isolated and pooled together. Isolated LDL was dialyzed against phosphate-buffered saline (PBS, pH 7.4) in the dark at 4°C, stored at 4°C and utilized within one week for functional assays. The chemical composition of isolated LDL was determined by enzymatic colorimetric tests using commercially available assays (Diasys, Germany)[7]. The rHDL particles were added to LDL directly before oxidation. LDL oxidation was induced by copper sulfate ($CuSO_4$, final concentration of 0.05 µM). The extent of LDL oxidation in the presence or absence of rHDLs was assessed using 2′, 7′-dichlorofluorescein diacetate (DCFH) fluorescent probe by Fluorescence Microplate Reader (Gemini, Molecular Devices, USA) as previously described[8]. The oxidability of rHDL particles alone was also measured, in the absence of LDL, in the incubations containing DCFH, $CuSO_4$ and rHDL at the concentrations indicated above.

Phospholipid transfer from LDL to rHDL.

The transfer of phospholipids from LDL to rHDL was evaluated using human LDL labeled with DiI (1, 1′-dioctadecyl-3, 3, 3′, 3′-tetramethylindocarbocyanine perchlorate) fluorescent probe as described previously for the lipolytic assay[9] with some modifications. Briefly, LDL was labeled with DiI probe (5 µM) at a DiI: LDL-phospholipid mass ratio of 1:13 via gentle stirring overnight at 37C°

in the presence of lipoprotein- deficient plasma (LPDP) as a source of lipid transfer proteins at a LPDP: LDL volume ratio of 1:100. The Dil-Labelled LDL was separated from unbound DiI by filtration through PD-10 Sephadex G-25 desalting column (GE HealthCare, US) and Dil-LDL filtrate was collected. Dil-LDL (7.5 mg phospholipid/dL) was mixed with rHDL (4 mg ApoA-I/dL), , 50µl of Tris buffer (0.4 M, PH 8) and PBS to a final volume of 200 µl, and the mixtures were incubated at 37C° for 1 hour to achieve phospholipid transfer ApoB-deficient plasma (1/30 v/v) obtained form an healthy donor was employed in parallel to incubation with rHDL as a reference and quality control[10]. The reaction was terminated by placing the mixture on ice, and Dil-rHDL-containing supernatants were isolated from each sample using ApoB-depleting precipitant (i.e. phosphotungstic acid, $MgCl_2$, NAOH; pH 6.2) as previously described[9]. The fluorescence intensity of Dil in rHDL was measured at an excitation wavelength of 550 nm and emission wavelength of 564 nm using Fluorescence Microplate Reader (Gemini, Molecular Devices, USA). The phospholipid transfer from LDL to rHDL was calculated via normalizing the fluorescence intensity in rHDL-containing supernatant to that of a reference normolipidemic HDL (employed as ApoB-deficient plasma) and presented as a percentage.

Western blotting analysis.

Total protein from THP-1 macrophages plated at a density of 4.0×10^6 cells/well into 6-well plates treated with different rHDLs (20 µg ApoA-I/ml) 4h, followed by 30 minutes stimulation with LPS, was extracted and analyzed by Western blotting as previously described[11]. Twenty micrograms of protein were separated by electrophoresis (2.5 hours, 120 Volt) on a 10% bis-Tris polyacrylamide gel and transferred onto a nitrocellulose membrane (overnight at 40 Volt; Cytiva Life Sciences, USA). Subsequently, the membrane was blocked, incubated with primary antibodies at 1:1000 overnight and revealed with IR dye 680RD and 800CW secondary antibodies at 1:10000 overnight (Li-COR, USA). Quantification of Western blots was performed using Li-Cor scanner (Odyssey system, Li-COR Biosciences, Germany). Phospho-SAPK/ JNK (Thr183/Tyr185) antibody (#9251), SAPK/JNK antibody

(#9252), phospho-p44/42 MAPK (Erk1/2) (Thr202/Tyr204) (D13.14.4E) XP® Rabbit mAb (#4370), p44/42 MAPK (Erk1/2) (137F5) Rabbit mAb (#4695), phospho-p38 MAPK (Thr180/Tyr182) (D3F9) XP® Rabbit mAb (#4511), and p38 mitogen-activated protein kinase (MAPK) antibody (#9212), were purchased from Cell Signaling Technology (Massachusetts, USA).

Eicosanoid quantification by LC/MS-MS.

The impact of rHDLs on the long-term eicosanoids production was investigated in THP-1 macrophages plated at a density of 4.0 × 10^6 cells/well into 6-well plates and treated with different rHDLs (20 µg ApoA-I/ml) for a period of 16h. At the end of the treatment, cells were detached with EDTA-trypsin and centrifuged, supernatant was aspirated and cell pellets were stored at -80C°.

Cell pellets (100 µL) were spiked with an internal standard mixture (5µl) containing 0.5, 0.5, 2, 2 and 0.5 ng of TxB2-d4, PgE2-d4, 13(S)HODE-d4, 9(S) HODE-d4 and 15(S)HETE-d8, respectively. Lipids were extracted with 400 µl of cold methanol containing Butylated Hydroxytoluene (BHT; 50 mg/L) as an anti oxydantfor 10 min at 0°C. After a centrifugation for 5 min at 4°C, and 15 000g, supernatants were collected and the pellets were reextracted as previously described[12]. Pooled supernatants were evaporated to dryness under vacuum. Dried extracts were finally solubilized with 50 µL of methanol/water (50/50 v/v).

High performance liquid chromatography (HPLC) was performed using Vanquish UPLC System equipped with an autosampler, a binary pump and a column oven (ThermoScientific, France). Lipids were separated on a Luna C18 3µm – 250 x 2 mm column (Phenomenex, France) maintained at 45°C. Mobile phases consisted of acetic acid 0.1 % in water (A) and acetonitrile (B). The flow rate was maintained constant at 0.3 ml/min. Before each new injection, the column was equilibrated with 25 % of the phase B for 5 min. For lipid species separation, the gradient was then ramped to 45 % B in 3 min, maintained at 45% B for 2 min, ramped to 60 % B in 5 min, then to 98 % B over the subsequent 6 min and finally maintained at 98 % B for the next 4 min. The LC system was coupled to an Altis Plus triple quadrupole mass spectrometer (ThermoScientific, France) equipped with an H-ESI electrospray

ionization source. Source vaporizer and ion transfer tube temperatures were set at 350°C and 325°C respectively.

Analysis was conducted in negative selected reaction monitoring mode (SRM) using the following transitions: PgE2 351.1 → 271.1 / PgE2-d4 355.1 → 275.1 (Collision energy (Coll) 16 V); TxB2 369.4 → 169.0 / TxB2-d4 373.3 → 173.1 (Coll 5 V); 13(S)HODE 295.3 → 195.0 / 13(S)HODE-d4 299.3 → 195.0 / 9(S)HODE 295.3 → 171.1 / 9(S)HODE-d4 299.3 → 172.0 (Coll 18 V); 15(S)HETE 319.2 → 219.0 / 15(S)HETE-d8 327.3 → 226.1 (Coll 12V); 11(S)HETE 319.2 → 167.2 (Coll 15V); 5(S)HETE 319.2 → 115.2 (Coll 14V).

Calibration curves were obtained using authentic standards, with the exception of 11(S) HETE and 15(S) HETE for which 5(S) HETE curve with 15(S) HETE-d8 as an internal standard was used. All standards were extracted using the same method as the samples. Linear regression was applied to calculate concentrations of eicosanoids in the samples.

References

1. Rye, K. A. Interaction of apolipoprotein A-II with recombinant HDL containing egg phosphatidylcholine, unesterified cholesterol and apolipoprotein A-I. *Biochim. Biophys. Acta* **1042**, 227–236 (1990).
2. Tanaka, N. *et al.* Eicosapentaenoic Acid-Enriched High-Density Lipoproteins Exhibit Anti-Atherogenic Properties. *Circ. J. Off. J. Jpn. Circ. Soc.* **82**, 596–601 (2018).
3. Guillas, I. *et al.* Identification of the specific molecular and functional signatures of pre-beta-HDL: relevance to cardiovascular disease. *Basic Res. Cardiol.* **118**, 33 (2023).
4. Camont, L. *et al.* Small, dense high-density lipoprotein-3 particles are enriched in negatively charged phospholipids: relevance to cellular cholesterol efflux, antioxidative, antithrombotic, anti-inflammatory, and antiapoptotic functionalities. *Arterioscler. Thromb. Vasc. Biol.* **33**, 2715–2723 (2013).
5. Folch, J., Lees, M. & Sloane Stanley, G. H. A simple method for the isolation and purification of total lipides from animal tissues. *J. Biol. Chem.* **226**, 497–509 (1957).
6. Ejsing, C. S. *et al.* Automated identification and quantification of glycerophospholipid molecular species by multiple precursor ion scanning. *Anal. Chem.* **78**, 6202–6214 (2006).
7. Zakiev, E. *et al.* Distinct phospholipid and sphingolipid species are linked to altered HDL function in apolipoprotein A-I deficiency. *J. Clin. Lipidol.* **13**, 468-480.e8 (2019).
8. Hussein, H. *et al.* Small, dense high-density lipoprotein 3 particles exhibit defective antioxidative and anti-inflammatory function in familial hypercholesterolemia: Partial correction by low-density lipoprotein apheresis. *J. Clin. Lipidol.* **10**, 124–133 (2016).
9. Ma, F. *et al.* Phospholipid transfer to high-density lipoprotein (HDL) upon triglyceride lipolysis is directly correlated with HDL-cholesterol levels and is not associated with cardiovascular risk. *Atherosclerosis* **324**, 1–8 (2021).

10. Kontush, A. *et al.* Preferential sphingosine-1-phosphate enrichment and sphingomyelin depletion are key features of small dense HDL3 particles: relevance to antiapoptotic and antioxidative activities. *Arterioscler. Thromb. Vasc. Biol.* **27**, 1843–1849 (2007).
11. Darabi, M. *et al.* Phosphatidylserine enhances anti-inflammatory effects of reconstituted HDL in macrophages via distinct intracellular pathways. *FASEB J. Off. Publ. Fed. Am. Soc. Exp. Biol.* **36**, e22274 (2022).
12. Ménégaut, L. *et al.* Profiling of lipid mediators in atherosclerotic carotid plaques from type 2 diabetic and non-diabetic patients. *Prostaglandins Leukot. Essent. Fatty Acids* **184**, 102477 (2022).

Supplemental Table 1.

Fasting plasma lipid levels	Median [Q1-Q3]
**Age (year)**	61 [53-66]
**Weight (kg)**	60.6 [54.8-72.73]
**BMI (kg/m^2)**	24.2 [21.2-28.65]
**Waist (cm)**	87.7 [87-97]
**HbA1c (%)**	5.8 [5.6-6.2]
**TC (mg/dL)**	230 [199-264]
**TG (mg/dL)**	95 [69-144.3]
**Fasting glucose (mmol/L)**	4.9 [4.47-5.4]
**LDL-C (mg/dL)**	147 [116.5-174.8]
**HDL-C (mg/dL)**	63 [53-74]
**ApoA-I (mg/dL)**	164 [149-187]
**ApoB (mg/dL)**	103 [93-121]
**Lp(a) (mg/dL)**	22 [9-59.7]
**c-IMT**	0.62 [0.54-0.75]
**Lipid-lowering therapy (%)**	57

Supplemental Table 1. Clinical data of female patients with metabolic syndrome (n=86)

Supplemental Table 2.

A.

rHDL	Composition/(Mole) ApoA-I	Soy-PC	Soy-PE	ARA-PE	EPA-PE	DB in 1 Mole PC	DB in 1 Mole PE	DB in total PL/1 Mole ApoA-I	% DB relative to Soy-PC rHDL
Soy-PC	1	90	-	-	-	1.54	-	139	-
Soy-PE	1	70	20	-	-	1.54	1.58	140	+ 0.72%
ARA-PE	1	70	-	20	-	1.54	2.50	158	+ 13.7%
EPA-PE	1	70	-	-	20	1.54	2.50	158	+ 13.7%

B.

rHDL	ApoA-I (mg/ml)	Phospholipid (mg/ml)	Phospholipid/apoA-I weight ratio
Soy-PC	5.86	10.6	1.82
Soy-PE	6.15	8.63	1.40
ARA-PE	5.58	8.06	1.44
EPA-PE	5.22	8.10	1.55

Supplemental Table 2. Phospholipid: protein ratio in rHDL particles and their double bond content. rHDL particles were prepared by mixing human ApoA-I and Soy-PC without or with either Soy-PE, ARA-PE (16:1/20:4) or EPA-PE (16:0/20:5). **A.** ApoA-I and phospholipid amounts used to prepare rHDL particles. **B.** ApoA-I and phospholipid composition of the rHDL particles measured after reconstitution. The number of double bonds was calculated using LS/MS-MS lipidomic data. ApoA-I: apolipoprotein A-I; DB: double bond; EPA: eicosapentaenoic acid; ARA: arachidonic acid; rHDL: reconstituted HDL; PC: phosphatidylcholine; PE: phosphatidylethanolamine.

Supplemental Table 3.

Primers			
Gene	Full Name	Forward (5'-3')	Reverse (3'-5')
HSP90AB1	heat shock protein 90kDa alpha (cytosolic), class B member 1	CTC TGT CAG AGT ATG TTT CTC GC	GTT TCC GCA CTC GCT CCA CAA A
NONO	non-POU domain-containing octamer-binding housekeeping gene	CAT CAA GGA GGC TCG TGA GAA G	TGG TTG TGC AGC TCT TCC ATC C
TUBA	α-tubulin	GAT GCT GCC AAT AAC TAT GCC CGA G	GAA AAC CAA GAA GCC CTG AAG ACG G
ABCA1	ATP-binding cassette A1	GCC AAG GAC CAA AGT GAT G	CAC CCC GTA TGA ACA GGA TT
ABCG1	ATP-binding cassette G1	CCG ACC GAC GAC ACA GAG A	CTG AGC ACG AGA CAC CCA CCA CA
SRB1	Scavenger receptor class B type 1	CAT CTA CCC ACC CAA CGA AG	TGA GGA AGT GAG GAT GGG AG
IL1β	Interleukin 1 beta	TCC AGG GAC AGG ATA TGG AG	TCA TCT TTC AAC ACG CAG GA

Antibodies	
**Target protein**	Cell Signaling Reference
Phospho-SAPK/ JNK (Thr183/Tyr185)	#9251
SAPK/JNK	#9252
phospho-p44/42 MAPK (Erk1/2) (Thr202/Tyr204)	#4370
p44/42 MAPK (Erk1/2) (137F5) Rabbit mAb	#4695
phospho-p38 MAPK (Thr180/Tyr182) (D3F9) XP® Rabbit mAb	#4511
p38 mitogen-activated protein kinase (MAPK) antibody	#9212

Supplemental Table 3. List of primers and antibodies used in this study.

Supplemental Table 4.

A.

PE (36:5) subspecies	Most probable µmol/l	Upper-limit µmol/l
1. PE(14:1/22:4)	0.000 – 0.004 µM	0.278 – 0.856 µM
2. PE(16:0/20:5)	0.453 – 0.865 µM	6.093 – 10.107 µM
3. PE(16:1/20:4)	0.111 – 0.385 µM	0.883 – 3.383 µM
4. PE(18:1/18:4)	0.000 – 0.008 µM	0.009 – 0.125 µM
5. PE(18:2/18:3)*	0.007 – 0.011 µM	0.109 – 0.225 µM
6. PE(18:2/18:3)*	0.032 – 0.040 µM	0.58 – 0.696 µM

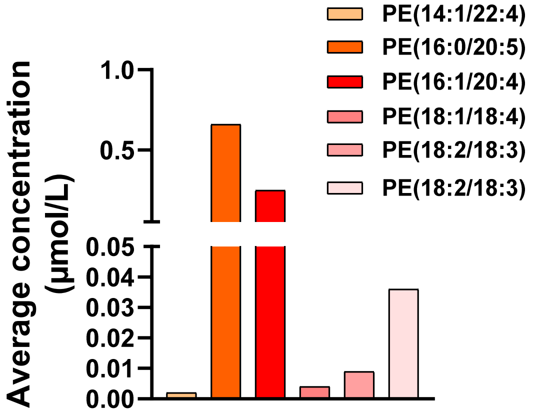

B.

Chemical structure of PE (36:5) subspecies

1. PE(14:1/22:4)

4. PE(18:1/18:4)

2. PE(16:0/20:5)

5. PE(18:2/18:3)

3. PE(16:1/20:4)

6. PE(18:2/18:3)

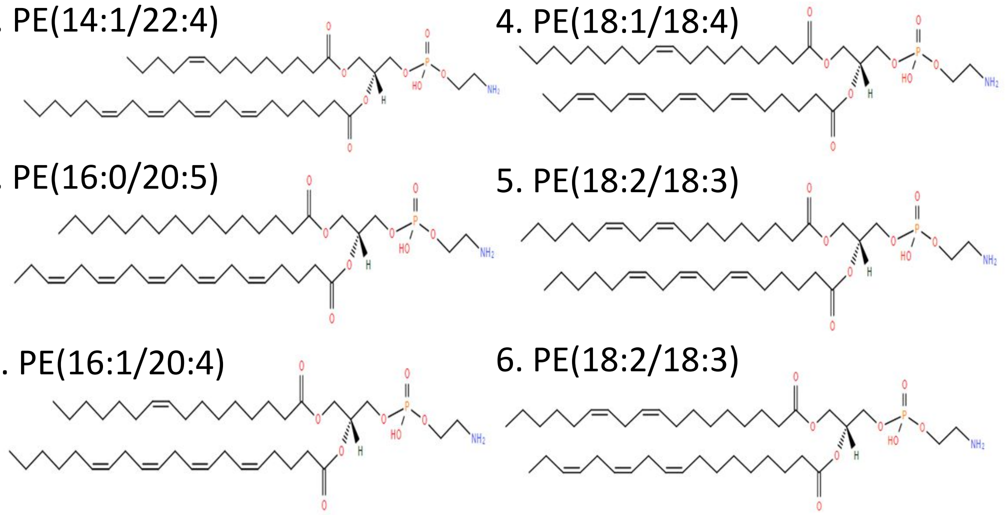

C14:1 = Myristoleic acid
C16:0 = Palmitic acid
C16:1 = Palmitoleic acid
C18:1 = Oleic acid
C18:2 = Linoleic acid
C18:3 = alpha-Linolenic acid
C18:3 = gamma-Linolenic acid
C18:4 = Stearidonic acid
C20:4 = Arachidonic acid
C20:5 = Eicosapentaenoic acid
C22:4 = Adrenic acid

Supplemental Table 4. Subspecies of PE (36:5) in human plasma. A. Blood concentrations of the six putative molecular subspecies of PE (36:5) predicted from the quantified individual fatty acid chains that construct these phospholipids in normolipidemic adults (both male + females). **B.** Chemical structure of PE (36:5) subspecies and the fatty acid moieties that constitute the six PE (36:5) subspecies. PE: phosphatidylethanolamine. *gamma-Linolenic (omega-6) vs alpha-Linolenic (omega-3) acid moiety.

Supplemental Figure 1.

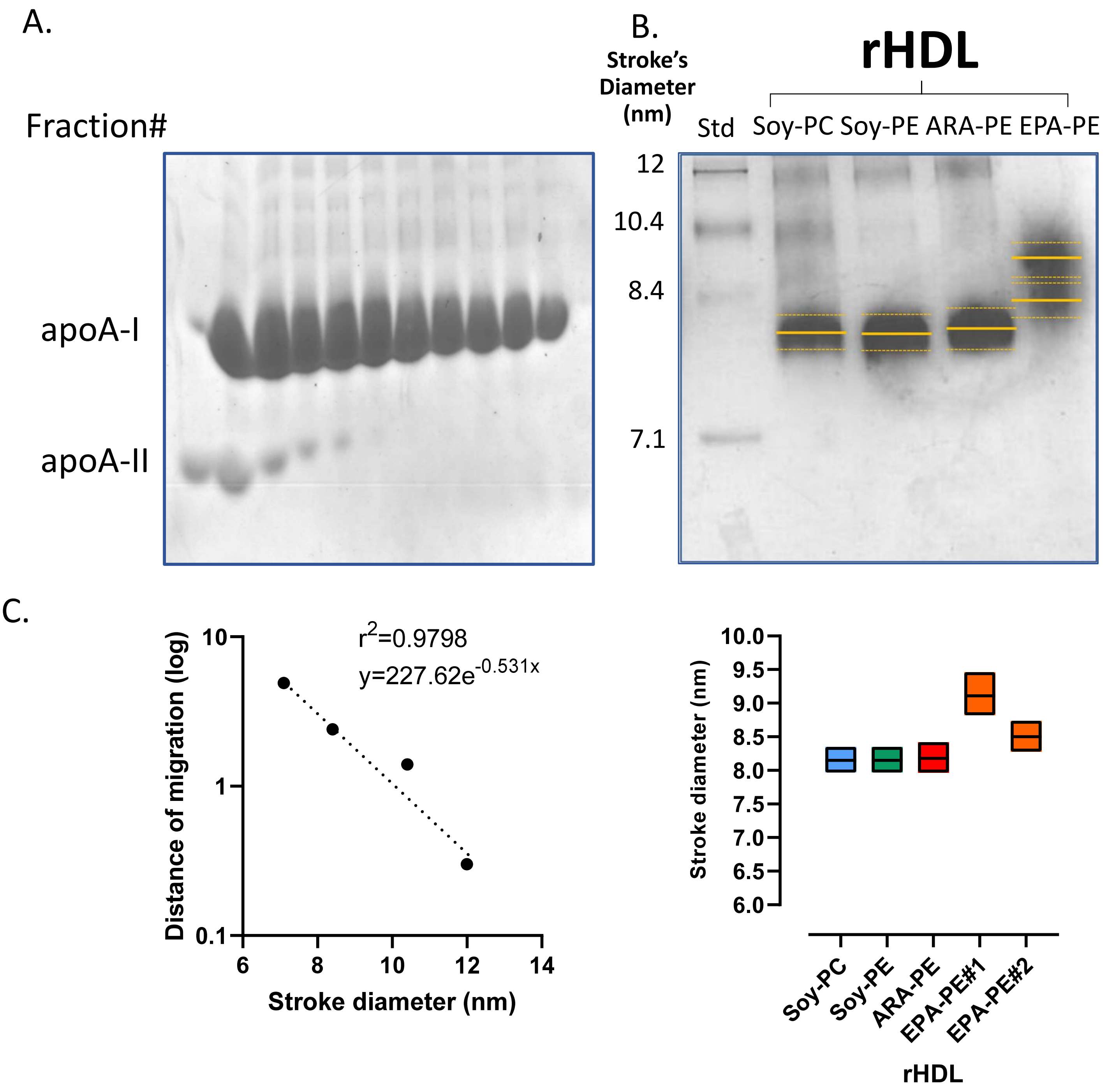

D.

	Soy-PC rHDL			Soy-PE rHDL			ARA-PE rHDL			EPA-PE rHDL		
	Diameter (nm)		%	Diameter (nm)		%	Diameter (nm)		%	Diameter (nm)		%
Band#1	11.94		6.9	11.94		5.7	11.94		4.7	9.28	±0.3	55
Band#2	9.63		10.6	8.06	±0.3	94.3	8.11	±0.22	95.3	8.43	±0.23	45
Band#3	8.11	±0.18	82.6									

Supplemental Figure 1. Quality control of ApoA-I and rHDL particles. A. Denaturing sodium dodecyl sulfate-polyacrylamide gel electrophoresis (SDS-PAGE) analysis to control the purity of ApoA-I containing fractions performed using 20% denaturing polyacrylamide gel revealed with Coomassie blue. **B.** Nondenaturing PAGE analysis to control the size of rHDLs performed on a native 4-30% TAE gradient gel revealed with Coomassie blue. Standard proteins of known diameters were used for the calibration. Calculated size **(C)** and amount **(D)** of rHDLs. ApoA-I: apolipoprotein A-I; ApoA-II: apolipoprotein A-II; EPA: eicosapentaenoic acid; ARA: arachidonic acid; rHDL: reconstituted HDL; PC: phosphatidylcholine; PE: phosphatidylethanolamine; Std: standard.

Supplemental Figure 2.

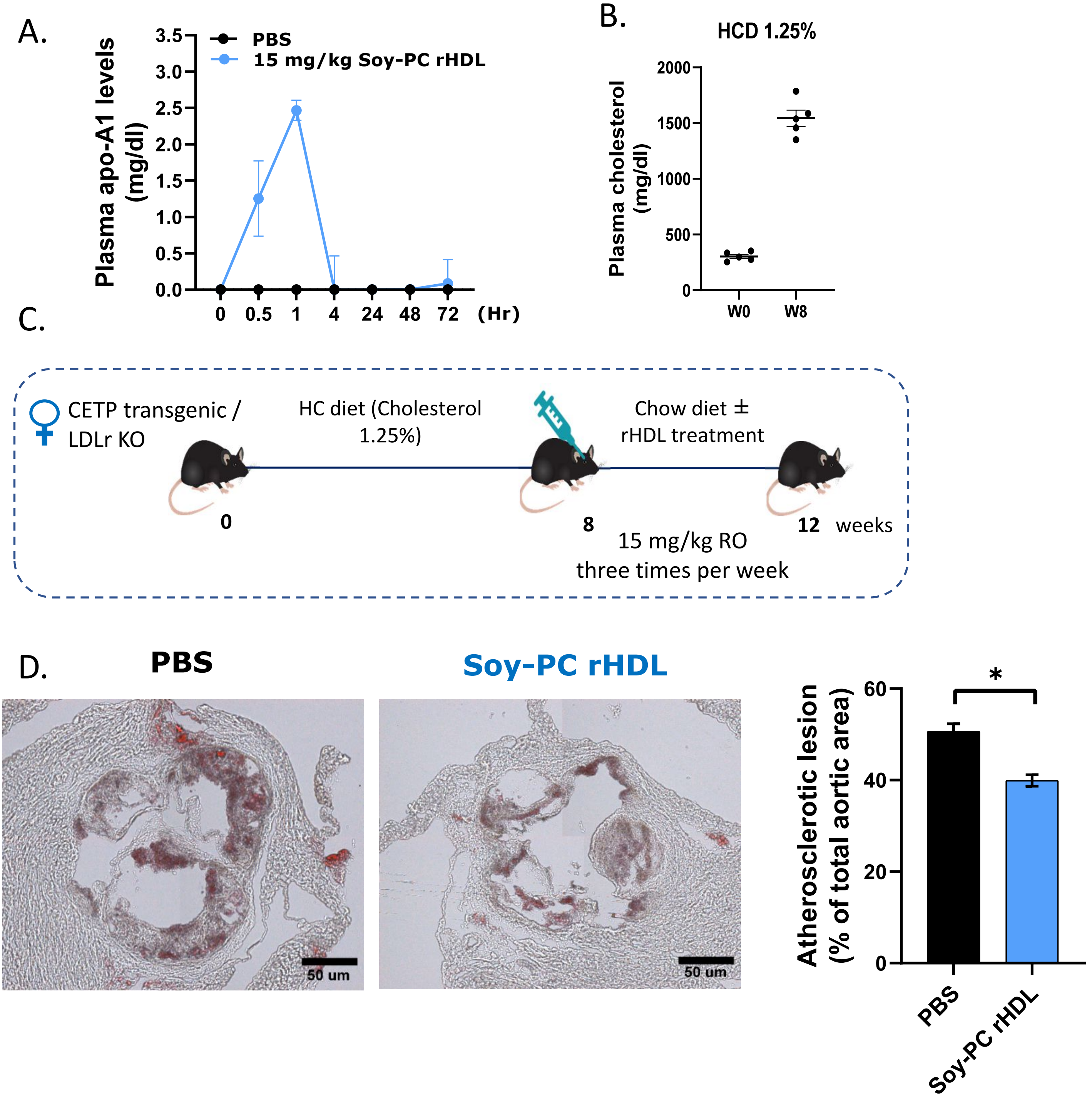

Supplemental Figure 2. Injection of Soy-PC rHDL reduced the size of aortic atherosclerotic plaque in CETP transgenic-LDLr -/- female mice. A. Plasma levels of human ApoA-I at various time points after a single injection of Soy-PC rHDL (n=3) or PBS (n=3) to validate it's effectiveness. **B.** Plasma cholesterol levels after 8 weeks of HCD diet to validate the effectiveness of HCD. **C.** CETP transgenic/ LDL receptor KO female mice were fed high cholesterol diet (HCD) for 8 weeks before injection with 15 mg/kg of Soy-PC rHDL (n=2) or PBS (n=2), retro-orbitally (RO), every second day. The mice received 10 injections of either treatment, and were maintained on chow diet during rHDL treatment. The hearts were carefully dissected at the level of the aortic root, and embedded in tissue-tek optimum cutting temperature (OCT) medium, then cut every 10 μm from the aortic root at the level of 3 valves. **D.** Sections were labeled with hematoxylin-eosin to quantify the atherosclerotic plaque size. The extent of the atherosclerotic plaque was quantified using ImageJ software. *$p<0.05$ vs Soy-PC rHDL. CETP: cholesteryl ester transfer protein; HC: high cholesterol; rHDL: reconstituted high density lipoprotein; PC: phosphatidylcholine; PBS: Phosphate buffered saline.

Supplemental Figure 3.

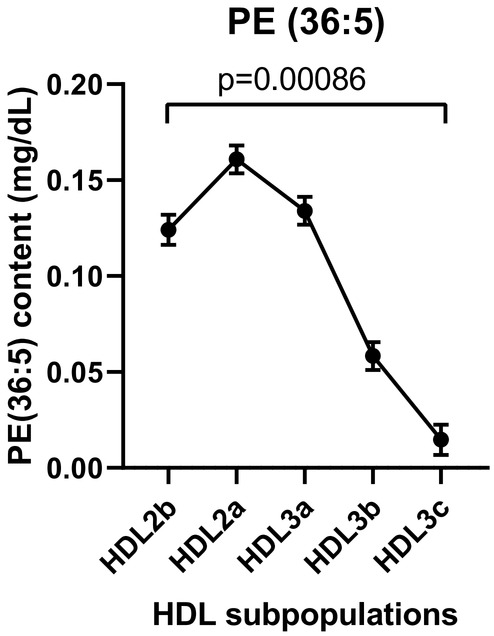

Supplemental Figure 3. PE(36:5) was mostly carried out by HDL2 subpopulations.
PE(36:5) content in HDL subspecies in 12 normolipidemic healthy individuals from the study by Camont et al.[4] Values are Mean±S.E.M. One-way ANOVA test.

Supplemental Figure 4.

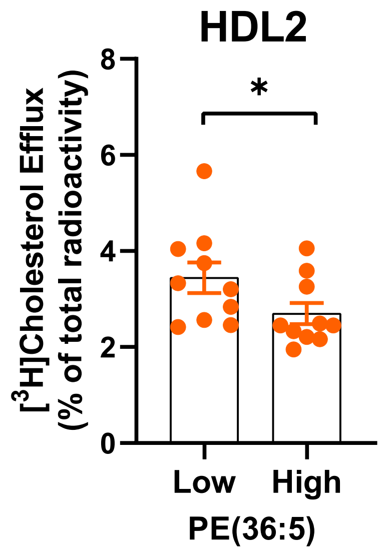

Supplemental Figure 4. Enriched PE(36:5)-HDL2 exhibited a reduced capacity to promote macrophage cholesterol efflux. The cholesterol efflux capacity of plasma HDL2 isolated from patients according to the PE(36:5) content (Low versus high, 10 patients per group) was determined using THP-1 macrophages loaded with radiolabeled [3H] cholesterol and incubated with 15µg/ml of HDL2-PL for 4 hours. Values are Mean±S.E.M., *p<0.05.

Supplemental Figure 5.

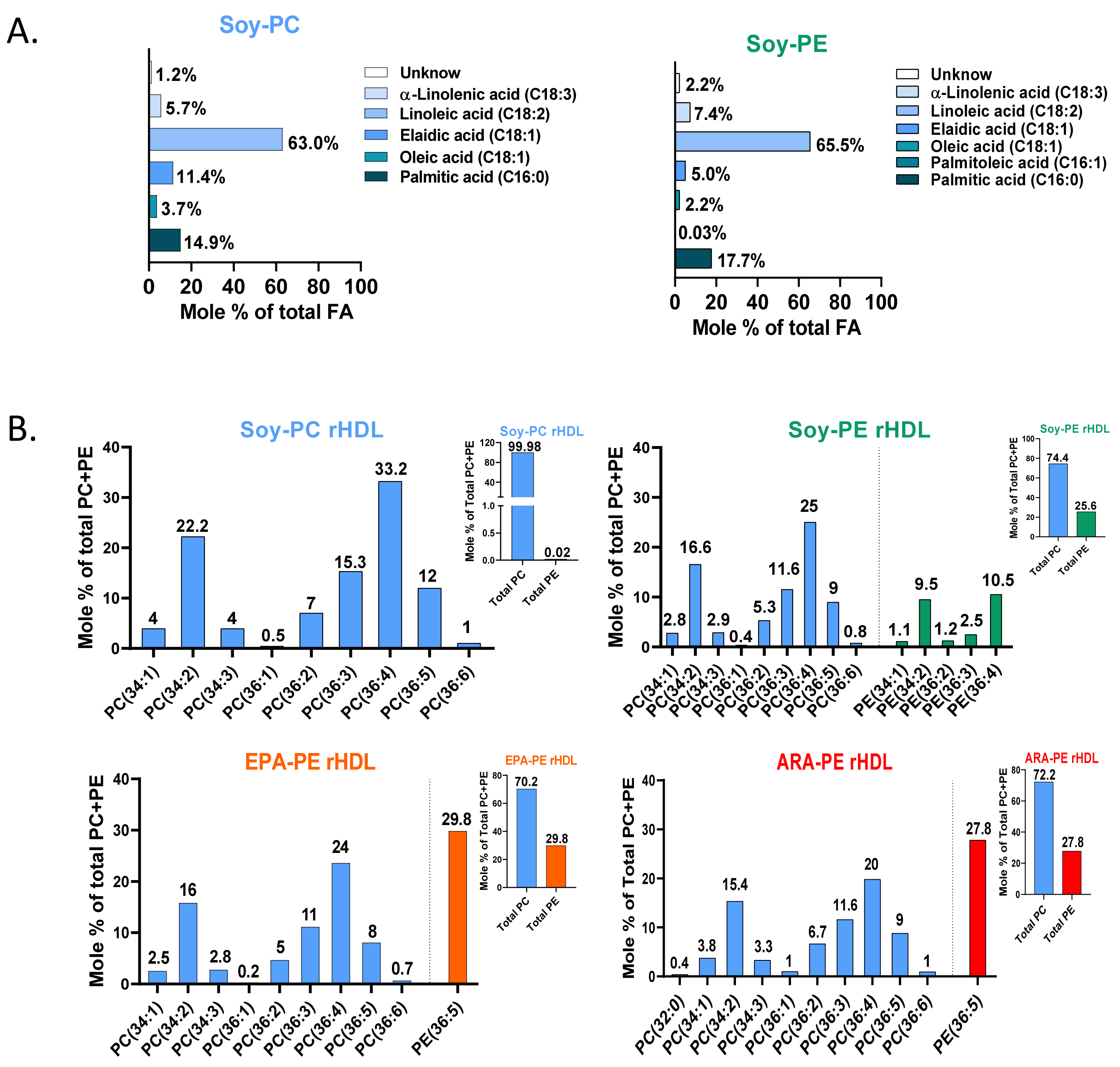

Supplemental Figure 5. Compositional analysis of rHDL formulations used in this study. A. Fatty acid composition of Soy-PC, and Soy-PE (Avanti Polar Lipids) that were used to produce rHDLs. Two forms of PE (36:5) including ARA-PE (PE (16:1/20:4)), and EPA-PE (PE (16:0/20:5)), synthetised by UMR5246 Institute, were also used to produce rHDLs. **B.** LC/MS/MS Lipidomic analysis of the rHDL formulations including Soy-PC rHDL, Soy-PE rHDL, ARA-PE rHDL, and EPA-PE rHDL. Data are shown for a representative preparation of rHDL. ARA: arachidonic acid ; EPA: eicosapentaenoic acid; FA: fatty acid; PC: phosphatidylcholine; PE: phosphatidylethanolamine.

Supplemental Figure 6.

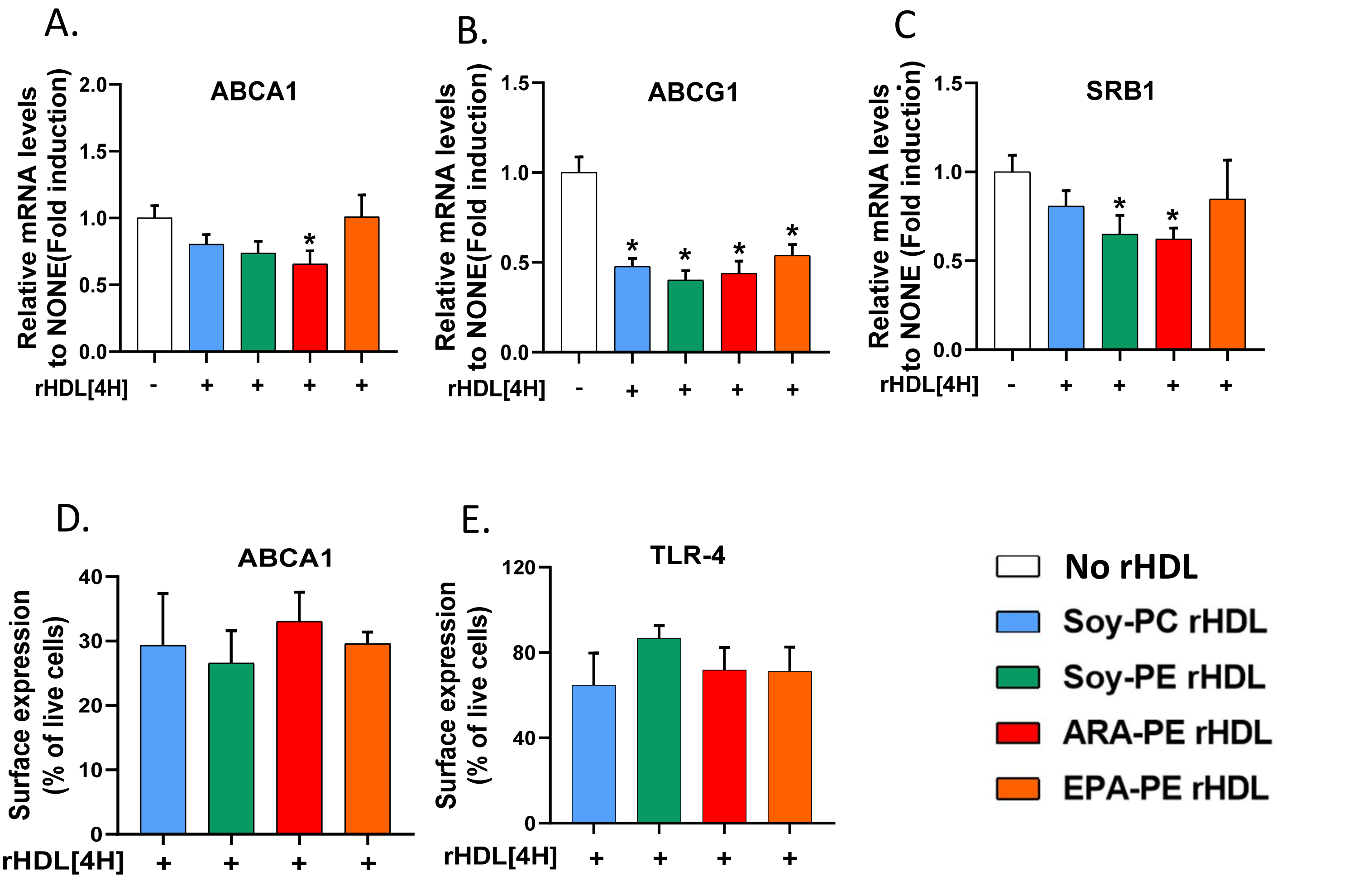

Supplemental Figure 6. The impact of rHDLs on the expression of the major genes involved in cholesterol efflux pathways as well as on the ABCA1 and TLR-4 surface expression. A-C. To evalute the impact of rHDLs on the expression of the major genes involved in cholesterol efflux, THP-1 cells were treated with 20 µg ApoA-1/ml of different rHDLs for 4 hours and the levels of ABCA1, ABCG1 and SRB1 mRNA were evaluated. **D.** To evaluate the impact of rHDLs on the surface expression of ABCA1 in THP-1 macrophages, cells treated with 20 µg ApoA-I/ml of rHDL for 4hours were collected and incubated with 100 µl of the ABCA1 rabbit primary antibody diluted in FcR blocking reagent containing PBS-FBS 5% (1:50 v/v) for 30 minutes. Then cells were stained with 100 µl of a secondary fluorescent antibody (Alexa-fluor 594 gout anti-rabbit IgG; 1:250 v/v) for additional 30 minutes. At the end of the incubation, cells were fixed using FOXP3 fixation diluent flow cytometry analysis. **E.** TLR-4 cell surface expression. *vs. no rHDL added, #vs. Soy-PC rHDL, ɸvs. Soy-PE rHDL, δvs. ARA-PE rHDL, with p values less than 0.05. Values are shown from at least 3 independent experiments that were performed in triplicate for each condition.

Supplemental Figure 7.

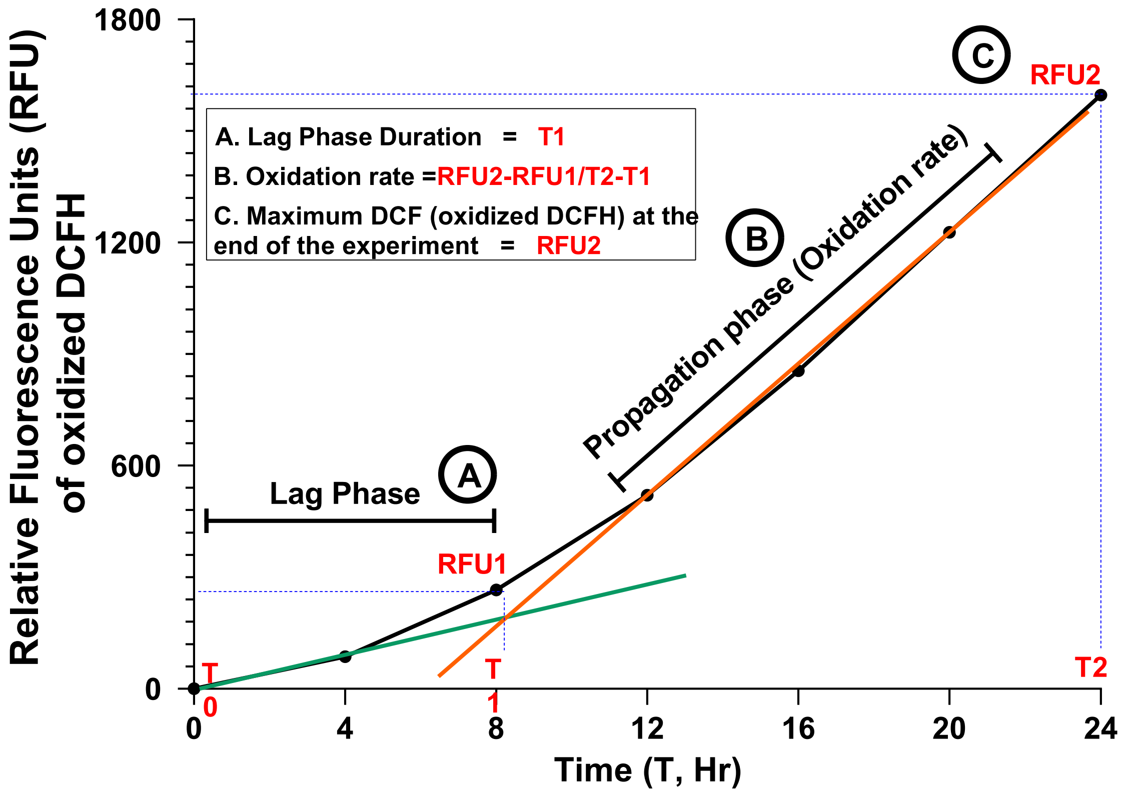

Supplemental Figure 7. Oxidation parameters calculated from biphasic oxidation kinetics to evaluate the extent of copper-induced LDL oxidation. The extent of LDL oxidation induced by copper ions was evaluated from the kinetic curve of the oxidation of dichlorofluorescin diacetate (DCFH) and apparition of DCF fluorescence measured during 24 hours, and expressed as relative fluorescence units (RFU). The kinetics displayed two characteristic phases, known as the lag and propagation phases. **A.** The lag phase represents a period of slow oxidation; its duration determined by extrapolating two lines: one originating from the curve's beginning (green line), and the other aligning the rapid increase in the oxidation (orange line). **B.** The propagation phase signifies a period of rapid oxidation that starts after the lag phase and continues to the end of the experiment. The slope observed during this phase reflects the oxidation rate. **C.** The final measurement of RFU indicates the accumulated maximum of DCF during the entire duration of the experiment. Shorter lag phase, higher oxidation rate in propagation phase and higher value DCF at the end of the experiment all indicate a higher level of LDL oxidation.
